# Cardiovascular Risk Determinants in Euthyroid Patients with Obesity: The Strange Case of TSH in Primary Prevention

**DOI:** 10.3390/jcm15041427

**Published:** 2026-02-11

**Authors:** Cristina Vassalle, Luisella Vigna, Paolo Piaggi, Marco Scalese, Laura Sabatino, Francesca Mastorci, Gabriele Trivellini, Filomena Napolitano, Francesca Gori, Alessandra Piontini, Alessandro Pingitore

**Affiliations:** 1 Fondazione G. Monasterio, Regione Toscana, 56124 Pisa, Italy; 2 Occupational Health Unit, Obesity and Work Center, EASO Collaborating Center for Obesity Management, Fondazione IRCCS Ca’ Granda Ospedale Maggiore Policlinico, 20122 Milan, Italy; 3 Department of Information Engineering, University of Pisa, 56122 Pisa, Italy; 4 Clinical Physiology Institute, Italian National Research Council (CNR), 56124 Pisa, Italy; scalese@ifc.cnr.it (M.S.);; 5 SS Laboratorio di Biochimica, SC Patologia Clinica, Fondazione IRCCS Ca’ Granda Ospedale Maggiore Policlinico, 20122 Milan, Italy; 6 Dipartimento di Anestesia, Cure Critiche e Emergenza, Fondazione IRCCS Ca’ Granda-Ospedale Maggiore Policlinico, 20122 Milano, Italy

**Keywords:** thyrotropin, euthyroid range, cardiovascular risk, subjects with obesity, ASCVD risk score

## Abstract

**Background:** Thyrotropin (TSH), even in the normal range, is associated with components of cardiometabolic syndrome. We aimed to assess the relation between TSH and cardiovascular (CV) risk in euthyroid patients with overweight/obesity without previous cardiac events. **Methods:** A total of 1588 subjects (1132 females, mean age 53 ± 14 years) were recruited. This was an observational study. TSH, body mass index, waist circumference (WC), creatinine, hepatic enzymes, homocysteine, C reactive protein, glycated hemoglobin, homeostatic model assessment for insulin resistance (HOMA-IR), basal and 2 h glucose and insulin, fibrinogen, uric acid, a complete blood count, a complete lipid profile, and blood pressure were measured in all subjects. The Atherosclerotic Cardiovascular Disease (ASCVD) risk score was calculated. **Results:** More severe degrees of obesity were associated with higher TSH quartiles; specifically, 33% of subjects with grade III obesity had TSH in the 75th percentile. Multiple regression showed that female gender (t-value 3.6, *p* < 0.001), HOMA-IR (1.9, ≤0.05) and aspartate transaminase (AST; 2.8, <0.01) represent independent determinant factors affecting TSH levels in the population at higher CV risk (intermediate–high ASCVD risk score > 7.5%; *n* = 709). Similarly, TSH determinants in subjects with central obesity (*n* = 1197, WC >102 cm males, >88 cm females) were female gender (2.2, <0.05), HOMA-IR (2.7, <0.01) and smoking habit (−2.3, <0.5). Moreover, there was no significant relationship between TSH and ASCVD risk score. **Conclusions:** Higher TSH levels in the euthyroid range are related to high degrees of obesity and some CV risk factors, in subjects at higher cardiometabolic risk; however, for the different weight and sign of CV determinants (e.g., smoking habit) on the TSH system, the ASCVD risk score cannot evidence this relationship.

## 1. Introduction

Thyroid hormones (THs) play a pivotal role in the homeostatic regulation of metabolism and other systems, including the cardiovascular (CV) system [1]. TH dysfunction can impair lipid and glycemic metabolism, promote endothelial dysfunction, induce a prolonged activation of the inflammatory system and increase oxidative stress, thereby potentially elevating CV risk [2].

Notably, a recent systematic review showed that low concentrations of thyroid-stimulating hormone (TSH) were associated with an increased risk of all-cause and CV mortality [3]. Moreover, TSH concentrations within the 60th–80th percentiles appeared to represent the optimal physiological range of thyroid function with respect to the risk of major CV events [3]. Moreover, obesity has also been associated with alterations in TSH levels, even within the conventionally defined euthyroid reference range [4]. It is otherwise noteworthy that age, gender or smoking are potential confounders of the relationship between obesity and TSH values [5,6,7]. Consistently, higher serum TSH concentrations were observed in Chinese women with obesity than in non-obese counterparts, while other authors identified female sex and smoking status as potential modulators of TSH levels in obesity [5,6]. Moreover, elevated TSH values, though still within the reference range, have been linked to several cardiometabolic risk factors [7]. Specifically, Meheran et al. found that high–normal TSH levels were associated with an increased risk of metabolic syndrome [8]. It is known that both subclinical and overt hyperthyroidism and hypothyroidism are related with CV risk factors [9]. In particular, hypothyroidism has been associated with dyslipidemia, hypertension, obesity and diabetes, whereas hyperthyroidism has been correlated with hypertension and blood hypercoagulability [9]. Nonetheless, from a pathophysiological point of view, a continuum of effects along the entire range of thyroid functioning, including the euthyroid range, is highly plausible [9]. However, although changes of thyroid function (within the euthyroid range) were found to be associated with CV risk factors, it is still unclear whether high–normal TSH within the normal range in euthyroid subjects may significantly affect the global atherosclerotic cardiovascular disease (ASCVD) risk [10]. The 2013 Atherosclerotic Cardiovascular Disease (ASCVD) Risk Score, developed by the American Heart Association (AHA) and the American College of Cardiology (ACC), is a validated tool used to estimate the 10-year risk of major adverse cardiovascular events (e.g., myocardial infarction, stroke, or death from coronary heart disease or stroke), by incorporating several key cardiometabolic risk factors [11].

In the present study, we aimed to evaluate the potential association between TSH levels within the normal range and various cardiometabolic risk factors, as well as the overall CV risk estimated using the ASCVD score. Our analysis was conducted in a primary prevention setting, including only patients without prior CV events. Furthermore, we focused on obese patients, as obesity itself is a CV risk factor and can negatively impact glucose and lipid metabolism, thus increasing overall CV risk.

## 2. Materials and Methods

### 2.1. Study Population

A total of 1588 adult Caucasian subjects with overweight/obesity (1132 females, mean age 53 ± 14, range 18–86 years) with TSH values within the reference range (<4.2 µU/mL), were included in the study. Patients were recruited at the Center of Obesity and Work, the occupational health unit of the work clinic “L. Devoto”, Fondazione IRCCS Ca’ Granda, Ospedale Maggiore Policlinico, Milan (Italy), where they were referred to a weight loss program. All data were assessed by history, laboratory or medical records; at enrollment, each participant underwent a medical examination, during which age, height, weight and body mass index (BMI), waist circumference (WC), and systolic and diastolic blood pressure (SBP and DBP) were recorded. Additionally, patients underwent blood withdrawal for laboratory assessment.

Overweight was defined as BMI between 25 and 29.9 kg/m^2^; obesity was defined as BMI ≥ 30 kg/m^2^; grade I obesity was defined as BMI 30–34.9 kg/m^2^; grade II obesity as BMI 35–39.9 and grade III as BMI ≥ 40 kg/m^2^. As BMI reflects total body mass relative to height, whereas WC is an index of abdominal fat, BMI categories were used to define overall weight status (from underweight to obese grades). Because this approach may lack the ability to discriminate between muscle and fat, WC is a better index of abdominal obesity (visceral fat).

The exclusion criteria included cardiac disease, thyroid diseases or thyroid replacement therapy, history of chronic disease or current malignancy and regular use of dietary supplements.

The 10 yr ASCVD risk was calculated using the following information: age, sex, race, total cholesterol (TotCH), high density lipoprotein (HDL) cholesterol, SBP, DBP, blood pressure lowering medication use, diabetes status, and smoking status. This was undertaken to estimate the 10-year risk of heart disease or stroke and to categorize individuals expressed as % according to low (<7.5%), intermediate (7.5–19%), and high risk (≥20%) [12].

People’s first language was used according to the standard recommendation of the European Association for the Study of Obesity (EASO), The Obesity Society (TOS), and Obesity Canada (OC) to reduce the bias and stigma associated with obesity [13].

The study was conducted according to the Good Clinical Practice guidelines and approved by the Human Ethic Committee of Fondazione IRCCS Ca’ Granda Ospedale Maggiore Policlinico (Registration number: 852). The study has been carried out in accordance with the World Medical Association Declaration of Helsinki.

### 2.2. Blood Samples and Laboratory Biomarkers

Blood samples were collected after an overnight fast (around 9 a.m.) and analyzed for a complete blood count (EDTA tubes; Advia 2120i, Siemens, Munich, Germany).

Moreover, glucose (sodium fluoride used as glycolysis inhibitor to preserve glucose), insulin (INS; EDTA tubes), triglycerides (TG), TotCH, HDL and low-density lipoprotein-cholesterol (LDL), gamma glutaryl transferase (GGT), aspartate transaminase (AST), alanine transaminase (ALT), creatinine, uric acid, glycated hemoglobin (HbA1c) and high-sensitivity C reactive protein (hsCRP) (lithium heparin tubes), fibrinogen (sodium citrate as the anticoagulant) and homocysteine levels (EDTA tubes placed immediately on ice to prevent artificial elevation by red blood cells). All analyses were performed at the Biochemical Laboratory of the Fondazione IRCCS Ca Granda -Ospedale Maggiore Policlinico (Milano, Italy), by routine automated laboratory instruments (Roche Diagnostics, Basel, Switzerland). Samples were centrifuged at 2500× *g* for 10 min (homocysteine centrifuged at 4 °C) and analyzed within a few hours of blood collection.

Insulin resistance (HOMA-IR) was evaluated with the homeostatic model assessment, calculated by multiplying fasting plasma insulin (FPI) by fasting plasma glucose (FPG), then dividing by the constant 22.5, according to the following formula: HOMA-IR = (FPI (mU/L) × FPG (mg/dL))/22.5 [14]. Metabolic syndrome (MS) was defined as the presence of at least three of the five ATP3 2005 criteria according to the National Cholesterol Education Program (NCEP) [15]:

1. Blood glucose > 100 mg/dL or drug treatment for elevated blood glucose;
2. HDL cholesterol < 40 mg/dL in men, <50 mg/dL in women or drug treatment for low HDL-C;
3. TG > 150 mg/dL or drug treatment for elevated triglycerides;
4. WC > 102 cm (men) or >88 cm (women);
5. Blood pressure > 130/85 mmHg or drug treatment for hypertension.

### 2.3. Statistical Analysis

Continuous variables were presented as mean ± standard deviation (SD) or median (interquartile range) based on normality of distribution, while categorical variables are presented as frequencies and percentages. The chi-squared (χ^2^) test was used to assess the association between two or more categorical variables. Log transformation was used for variables with high skewness to improve the performance of statistical models; back-transformed results were then used for data presentation. The unpaired Student’s *t*-test or Mann–Whitney U test were used to assess a significant difference between the means of two groups. The ANOVA test was used to compare the means of multiple groups to determine whether there was a statistically significant difference between them. Pearson correlation coefficients were used to assess relationships between continuous variables, *p* value was adjusted for multiple testing using false discovery rate (FDR) correction. A multivariate regression analysis was performed to evaluate the independent predictors of the dependent variable, specifically TSH levels. Linear and logistic regression models, adjusted for all confounding factors, were made using stepwise forward selection. Logistic regression analysis was used to assess the relationship between INS, 2hINS and HOMA-IR and a binary dependent variable (subjects in the highest TSH quartile or not) after multivariate adjustment.

Analyses were performed with the Statview statistical package, version 5.0.1 (SAS Institute, Abacus Concepts, Berkeley, CA, USA). A *p*-value of <0.05 was considered statistically significant.

## 3. Results

### 3.1. Characteristics of the Study Groups

Demographic and laboratory characteristics according to normal/overweight and obesity grades are shown in Table 1 (ANOVA test for numerical variables and χ^2^ test for categorical variables). Several parameters related to CV risk increased progressively with obesity severity, including age, blood pressure, and the levels of TSH, creatinine, GGT, ALT, AST, homocysteine, hsCRP, TG, LDL, HbA1c, HOMA-IR, basal and 2 h glucose and INS, fibrinogen, uric acid, white blood count (WBC), neutrophils (N), monocytes (M), and lymphocytes (L), whereas HDL decreased. Instead, there was no appreciable difference in terms of age, platelets (PLTs) and TotCH between the groups. TSH levels increased according to obesity grades (*p* < 0.05) (Table 1; ANOVA test), but not according to the presence of MS (1.8 ± 0.84 vs. 1.8 ± 0.86; unpaired *t*-test). Moreover, the proportion of subjects with TSH in the 75th percentile increased with obesity grades (23, 23, 26 and 33% in overweight, I, II and III grade of obesity, *p* < 0.05, respectively; χ^2^ test).

CV risk, assessed using the ASCVD score, was directly related to obesity severity, rising from 8.7% in overweight individuals to 13.4% in those with severe obesity (Table 1; χ^2^ test). Furthermore, the number of subjects at high ASCVD risk (≥20%) was significantly higher among individuals with severe obesity (11, 16 and 25%, *p* < 0.001 in overweight, grade I, II and III obesity, respectively; χ^2^ test).

### 3.2. Relationship Between TSH and Cardiometabolic Risk Factors and Score

Regression analysis (Table 2) revealed no significant association between TSH levels and the ASCVD score. Conversely, TSH values showed a significant positive correlation with fasting and 2 h post-load insulin, HOMA-IR, AST and BMI, though this was weak. In fact, following adjustment of type 1 error using FDR correction, these variables were no longer significant. As expected, the percentage of subjects in the highest TSH percentiles was greater among women than among men (305 [27%] vs. 89 [19%], *p* < 0.001).

Subjects in the highest TSH quartile showed significantly higher INS (15 ± 13.6 vs. 16.4 ± 11.4 mU/L, *p* < 0.01), 2hINS (59 ± 49 vs. 66 ± 56 mU/L, *p* < 0.05), and HOMA-IR (3.8 ± 4.6 vs. 4.2 ± 3.5, *p* < 0.01) than those in the lower quartile; these associations remained significant after adjusting for BMI, sex and age in the logistic regression models (INS OR = 1.91, 95%CI 1.15–3.17, *p* < 0.05; INS 2 h OR = 1.55, 95%CI 1.05–2.29, *p* < 0.05; HOMA-IR OR = 1.71, 1.09–2.69, *p* < 0.05).

Smokers had lower TSH levels than non-smokers (1.7 ± 0.8 vs. 1.8 ± 0.9 µU/mL, *p* < 0.01), and, among smokers, TSH values progressively decreased with the number of cigarettes smoked per day (r = −0.130, *p* < 0.05). In multivariate regression analysis, female sex, HOMA-IR, and AST levels were identified as independent determinants of TSH levels in subjects at higher cardiometabolic risk (intermediate-to-high ASCVD > 7.5%; n = 709) (Table 3). Similarly, when ASCVD was associated with WC (> 102 cm in Males M, >88 cm in females F; n = 1197), female gender, HOMA-IR and AST levels and smoking habit emerged as independent risk factors for higher cardiometabolic risk assessed as (Table 4).

## 4. Discussion

The results showed that higher levels of TSH in the so-called euthyroid range are associated with several risk factors, primarily with more severe degrees of obesity. These findings are consistent with previous evidence obtained from large population-based studies [7]. Regarding the relationship between thyroid function and obesity, the cause of the increased TSH levels observed in obesity remains unclear. Several hypotheses have been proposed, including the development of TH resistance in peripheral tissues in obese subjects. In fact, it was observed that the expression of TSH receptors is reduced in the adipose tissue of obese individuals [16,17]. TSH receptors and THs in adipose tissue play a crucial role in regulating cell proliferation and differentiation, a process regulated by the fine balance between lipogenesis, lipolysis and fatty acid oxidation [18,19]. Interestingly, Ortega et al. showed that the expression and activity of type I iodothyronine deiodinase (D1), the enzyme that produces triiodothyronine (T3) through the deiodination of thyroxine (T4), is increased in the subcutaneous and visceral adipose tissue of obese subjects [20]. The increase of D1 is associated with elevated leptin levels, which stimulate the hypothalamic–pituitary–thyroid (HPT) axis and consequently increase the secretion of thyrotropin-releasing hormone (TRH) and TSH [18]. The reversibility of these alterations in TSH and TH levels among individuals with obesity suggests the presence of an adaptive regulatory mechanism. In line with obesity-related data, individuals in the highest quartile of TSH showed higher fasting insulin, 2 h post-load insulin, and HOMA-IR values, indicating a predisposition to insulin resistance. Consistently, TH promotes proinsulin gene expression through activation of the PI3K-AKT pathway, triggering downstream events regulating key insulin-mediated metabolic processes, including glycogen synthesis, glucose uptake, and gluconeogenesis [21,22,23]. In particular, TH-mediated activation of the PI3K-AKT signaling pathway also exerts cardioprotective effects, encompassing antiapoptotic and antifibrotic actions, mitochondrial preservation, as well as stimulation of cell growth, differentiation, and neoangiogenesis [24,25,26]. These findings suggest that higher TSH levels, even within the reference range, may contribute to increased metabolic risk. In this context, we identified AST as an important determinant of TSH levels in individuals at elevated cardiometabolic risk. The liver–thyroid axis is characterized by a bidirectional relationship: hepatic function influences TH activation, inactivation, transport, and metabolism, whereas thyroid dysfunction can influence hepatocyte activity and promote liver fibrosis. Previous data has shown that TSH and AST levels often correlate in obese patients, reflecting increased liver fat and metabolic dysfunction [27]. In a large-scale study (> 10 000 adult Caucasian subjects), a progressive increase in AST and other liver enzymes (ALT and GGT) was correlated with TSH increase [28]. Moreover, a significant relationship between AST and TSH has been reported in young patients with liver disease [29]. These interactions emphasize the importance of a comprehensive endocrinological and gastroenterological evaluation, even in patients with TSH values within the normal reference range [30,31].

When ASCVD score was evaluated, its value increased significantly from overweight individuals to those with severe obesity. Among participants with a high ASCVD score (>20%), the proportion of subjects with severe obesity was significantly higher than in the other groups. However, no association was observed between ASCVD score and TSH levels, even when higher TSH percentiles were considered. The absence of a relationship between TSH and the ASCVD risk score represents a novel finding, suggesting a dual role of TSH. On the one hand, TSH values within the reference range are correlated with major CV risk factors, namely obesity and insulin resistance, which, in turn, are associated with the severity of ASCVD score. On the other hand, other relevant ASCVD components show an opposite pattern of association with TSH. Therefore, because the ASCVD score is an integrated variable, the overall effect of thyroid function on CV risk likely depends on the relative impact and direction (e.g., sex and smoking status) of its individual determinants. For example, although TSH levels were positively associated with obesity severity and HOMA-IR, they were inversely associated with smoking and male sex, both established CV risk factors. This opposing pattern may attenuate or obscure the overall relationship between circulating TSH levels and estimated CV risk. Consistent with previous evidence, we confirmed that smokers showed lower TSH concentrations, with a gradual increase observed after smoking cessation [32]. These alterations in TSH may also influence its association with waist circumference. The mechanisms by which smoking affects TSH levels remain incompletely understood and likely involve multiple pathways. Possible explanations include the effects of thiocyanate on thyroid autoimmunity, as well as increased sympathetic activity and increased type 2 iodothyronine deiodinase (D2) activity in smokers [33]. Furthermore, women have been shown to exhibit higher TSH levels than men. Epidemiological data consistently demonstrate a higher prevalence of thyroid disorders among women, whereas both experimental and clinical studies suggest a modulatory role of estrogens on thyroid function [34]. Experimental evidence also indicates that estradiol can stimulate leptin secretion, which in turn promotes TSH release, likely contributing to the sex-related differences in TSH levels observed in our cohort [35].

### Values and Limitations of the Study

This study has several limitations. Its single-center design and cross-sectional nature may limit the generalizability of the findings. Nevertheless, the large study population ensured adequate representation across all obesity categories. Future longitudinal studies with long-term follow-up data are needed to better define the relationship between thyroid function and major adverse CV events.

The lack of a non-obese control group is another limitation that should be addressed in future research. Moreover, only a single thyroid measurement was available, whereas repeated assessments over time would have provided a more accurate evaluation of potential changes in TH metabolism. Additionally, only TSH data were available as an index of hypothalamic–pituitary–thyroid axis function. Measuring THs, specifically free triiodothyronine (FT3) and free thyroxine (FT4), and thyroid autoantibodies (not available in our cohort) would have allowed for a more robust and comprehensive assessment of thyroid function, given their established relevance to metabolic and CV risk stratification. Specifically, this suggests a potential for misclassification within euthyroid range physiology that could track with obesity/insulin resistance and affect associations. However, the availability of TSH measurements reflects the clinical “real life”, where FT4 (and possibly FT3) are measured only when TSH is not normal.

Although several other CV risk assessment tools are available, we chose to use the ASCVD risk score, due to its prior validation in obese populations [36], indicating the appropriateness of its application in our study population, and given that the study findings may vary depending on the specific risk score applied. The ASCVD score provides a more accurate estimate of CV risk in individuals aged 40–79 years, who represented more than 80% of our study population. Nevertheless, we also applied this score to a small proportion of participants (<20%) outside this age range in order to include the entire cohort. Importantly, when we restricted the analysis to subjects aged 40–79 years, the association between TSH and ASCVD risk remained inconsistent, yielding overlapping results. In fact, the main finding evidenced by our study is that TSH does not show a clear association with traditional risk scores (e.g., ASCVD). This lack of correlation is likely attributable to the heterogeneous magnitude and direction of CV risk determinants and their complex interaction with the TH system, which may not be fully captured by conventional global CV risk indices. There are some relationships evidenced between TSH and some parameters that can reflect subtle alterations in individual CV risk factors. However, after FDR correction, these associations lost statistical significance, showing that none of the tested effects are strong enough in our population. Thus, associations that do not remain significant after FDR correction are therefore more appropriately described as exploratory trends. However, though the relationships among the various variables were weak, it should be considered that, in addition to TSH levels within the normal range, the values of all of the other considered parameters were also normal, or only mildly altered, in our population, which was in a stage of primary prevention. In fact, parameters were, in the majority/totality of subjects, within the reference range of the biomarker considered, an aspect which may contribute to reducing the observed correlation between two variables (see Table 1).

Finally, a post hoc power analysis was conducted using G*Power 3.1.2. Assuming an alpha level of 0.05, the sample size used in this study, and an effect size of 0.1, the calculated statistical power was 0.98.

## 5. Conclusions

The results demonstrate a relative increase in TSH levels among individuals with obesity and suggest a potential association between elevated TSH and CV risk, although no clear correlation was observed with the traditional ASCVD score. Specifically, among subjects with intermediate-to-high cardiometabolic risk (ASCVD risk score > 7.5%; n = 709), we found that female sex, insulin resistance (HOMA-IR), and liver function (AST) independently predicted TSH levels in the multivariate analysis; this information would be helpful for designing larger confirmatory trials. However, the lack of correlation with the global risk score may reflect the different magnitude and direction of CV risk determinants and the TH system, which may not be fully captured by conventional global CV risk indices. Therefore, because ASCVD incorporates multiple variables that may be differentially associated with TSH, the lack of a direct association between TSH and ASCVD should be interpreted with caution. The absence of a relationship between ASCVD and TH values should finally be viewed as a complementary observation rather than a mechanistic conclusion. The present data suggest further hypothesis-generating insights to better assess the impact of thyroid dysfunction—ranging from mild alterations to overt disorders—on CV outcomes, through the development specific and ad hoc risk assessment tools, as well as to better elucidate the importance of maintaining euthyroidism, even in cases of subclinical thyroid dysfunction. Currently, in hypothyroidism, levothyroxine (L-T4) replacement therapy is recommended when TSH levels exceed 10 mIU/L, typically initiated at a dose of 20–25 μg/day [36]. However, earlier preventive strategies, such as longitudinal monitoring of TSH levels, might be advisable at lower or higher TSH thresholds to prevent the development of cardiometabolic disorders and reduce the risk of adverse CV events in individuals with increased susceptibility, such as those who are obese. Further data are needed to confirm this hypothesis.

## Figures and Tables

**Table 1:** Demographic and laboratory data of 1588 subjects with overweight and obesity.

Variables	Overall Population (n = 1588)	Overweight (n = 402)	Grade I Obesity (n = 630)	Grade II Obesity (n = 362)	Grade III Obesity (n = 194)	*p* Value
Age (years)	52 ± 14	52 ± 14	53 ± 13	53 ± 14	52 ± 13	ns
Females/males	1132/456 (71/29)	311/91 (77/22)	437/193 (69/31)	249/113 (69/31)	135/59 (70/30)	<0.05
WC (cm)	102.3 ± 12.9	90.5 ± 7.9	100.5 ± 8.9	109.2 ± 9.6	119.3 ± 11	<0.001
SBP (mmHg)	127 ± 15	122 ± 14	126 ± 15	129 ± 15	134 ± 16	<0.001
DBP (mmHg)	79 ± 9	77 ± 9	78 ± 9	80 ± 9	83 ± 10	<0.001
Creatinine (mg/dL)	0.8 ± 0.3	0.78 ± 0.15	0.79 ± 0.18	0.84 ± 0.53	0.76 ± 0.16	<0.01
AST (IU/L)	20 (16–24)	19 (16–23)	19 (16–24)	19 (16–24)	21 (17–27)	<0.01
ALT (IU/L)	22 (16–31)	19 (15–28)	22 (16–30)	23 (17–32)	25 (19–37.5)	<0.001
GGT (U/L)	19 (14–30)	16 (11–25)	19 (14–30)	22 (16–33)	23 (17–34)	<0.001
Homocysteine (µmol/L)	10.7 (8.9–13.1)	10.3 (8.5–12.6)	10.8 (9–13.2)	11 (9.1–13.25)	11 (9–13.6)	<0.001
hs-CRP (mg/dL)	0.28 (0.14–0.55)	0.18 (0.1–0.33)	0.26 (0.13–0.49)	0.43 (0.23–0.7)	0.53 (0.24–0.9)	<0.001
TotCH (mg/dL)	211 ± 40.4	214 ± 40.6	211 ± 39.4	211 ± 42	204 ± 40.1	ns
HDL-C (mg/dL)	58 ± 15	64 ± 16	58 ± 15	55 ± 13	55 ± 13	<0.001
TG (mg/dL)	106 (78–142)	92.5 (69–123)	104 (77–146)	118 (86–163)	110.5 (87–138)	<0.001
LDL-C (mg/dL)	133 ± 35	133 ± 36	133 ± 34	135 ± 35	130 ± 36	ns
HbA1c (%)	5.8 (5.5–6.1)	5.7 (5.5–6)	5.8 (5.5–6)	5.9 (5.6–6.1)	5.9 (5.7–6.2)	<0.001
Fasting glucose (mg/dL)	94 (87–103)	91(85–98)	94 (87–103)	95.5 (89–106)	98 (90–110)	<0.001
Glucose-2 h (mg/dL)	100 (90–113)	96 (88–107)	100 (90–113)	101 (93–117)	108 (95–124)	<0.001
Fasting INS (mU/L)	12.7 (8.8–18.3)	9.1 (6.6–13.1)	12.4 (9–17.4)	15 (10.7–21.5)	18.8 (14–27.3)	<0.001
Insulin-2 h (mU/L)	47.3 (28.6–75.8)	36.4 (23–58)	45 (27.7–69.3)	53 (33.5–86.9)	70 (43.1–113)	<0.001
HOMA-IR	3 (2–4.6)	2 (1.5–3)	2.9 (2.1–4.3)	3.5 (2.5–5.4)	4.7 (3.4–7.5)	<0.001
Uric acid (mg/dL)	5.2 ± 1.3	4.8 ± 1.2	5.2 ± 1.3	5.5 ± 1.4	5.6 ± 1.3	<0.001
Fibrinogen (mg/dL)	328 (290–367)	314 (277–350)	320 (288–356)	340 (300–380)	363.5 (315–404)	<0.001
WBC (×10^9^/L)	6.8 ± 1.7	6.5 ± 1.6	6.7 ± 1.6	7.1 ± 1.7	7.7 ± 1.9	<0.001
N (×10^9^/L)	4 ± 1.4	3.7 ± 1.3	3.9 ± 1.3	4.2 ± 1.4	4.7 ± 1.6	<0.001
L (×10^9^/L)	2 ± 0.6	2 ± 0.5	2.1 ± 0.5	2.1 ± 0.6	2.2 ± 0.6	<0.001
M (×10^9^/L)	0.5 (0.37–0.59)	0.45 (0.36–0.58)	0.46 (0.378–0.57)	0.5 (0.39–0.62)	0.53 (0.41–0.61)	<0.001
PLT (×10^9^/L)	250 ± 60	249 ± 54	248 ± 61	249 ± 63	255 ± 60	ns
TSH (µU/mL)	1.81 ± 0.9	1.78 ± 0.8	1.76 ± 0.9	1.83 ± 0.9	2 ± 0.8	<0.05
10-year ASCVD risk (%)	11.2 ± 12.3	8.7 ± 10.6	10.8 ± 11.8	13.5 ± 14	13.5 ± 12.8	<0.001
MS	804 (51)	298 (74)	314 (50)	128 (35)	64 (33)	<0.001

Values are mean ± SD, median (interquartiles), or number (%). WC, waist circumference; SBP, systolic blood pressure; DBP, diastolic blood pressure; ALT, alanine aminotransferase; AST, aspartate aminotransferase; GGT, gamma-glutamyl transferase; hs-CRP, high-sensitivity C-reactive protein; TotCH, total cholesterol; HDL-C, high density lipoprotein cholesterol; TG, triglyceride; LDL-C, low density lipoprotein cholesterol; HbA1c, glycosylated hemoglobin; HOMA-IR, homeostatic model assessment for insulin resistance; WBC, white blood cells; N, neutrophils; L, lymphocytes; M, monocytes; PLT, platelets, TSH = thyroid-stimulating hormone; MS = metabolic syndrome. ns = not significant.

**Table 2:** Correlation between TSH and cardiometabolic parameters and risk.

	TSH	
Parameter	r	*p* Value (FDR)
Age (years)	−0.018 (−0.067–0.031)	0.472 (ns)
Weight (kg)	0.029 (−0.020–0.078)	0.243 (ns)
BMI (kg/m^2^)	0.065 (0.015–0.113)	0.010 (ns)
WC (cm)	0.027 (−0.023–0.078)	0.288 (ns)
ASCVD risk score	−0.013 (−0.064–0.037)	0.601 (ns)
SBP (mmHg)	−0.010 (−0.059–0.039)	0.687 (ns)
DBP (mmHg)	−0.009 (−0.058–0.040)	0.714 (ns)
Creatinine (mg/dL)	0.031 (−0.018–0.080)	0.217 (ns)
AST (IU/L)	0.058 (0.009–0.107)	0.021 (ns)
ALT (IU/L)	0.004 (−0.046–0.053)	0.882 (ns)
GGT (U/L)	−0.033 (−0.082–0.016)	0.186 (ns)
Homocysteine (µmol/L)	−0.008 (−0.057–0.042)	0.760 (ns)
hs-CRP (mg/dL)	0.015 (−0.034–0.065)	0.541 (ns)
TotCH (mg/dL)	0.029 (−0.020–0.078)	0.249 (ns)
HDL-C (mg/dL)	0.021 (−0.029–0.070)	0.413 (ns)
TG (mg/dL)	0.033 (−0.016–0.082)	0.183 (ns)
LDL-C (mg/dL)	0.031 (−0.019–0.080)	0.223 (ns)
HbA1c (%)	−0.013 (−0.085–0.060)	0.730 (ns)
Fasting glucose (mg/dL)	−0.013 (−0.062–0.036)	0.604 (ns)
Glucose-2 h (mg/dL)	−0.001 (−0.050–0.049)	0.977 (ns)
Fasting INS (mU/L)	0.059 (0.010–0.108)	0.019 (ns)
Insulin-2 h (mU/L)	0.049 (0.001–0.098)	0.049 (ns)
HOMA-IR	0.048 (0.001–0.097)	0.048 (ns)
Uric acid (mg/dL)	0.016 (−0.033–0.065)	0.522 (ns)
Fibrinogen (mg/dL)	−0.017 (−0.067–0.033)	0.498 (ns)
WBC (×0^9^/L)	0.01 (−0.039–0.060)	0.678 (ns)
N (×10^9^/L)	−0.003 (−0.053–0.046)	0.892 (ns)
L (×10^9^/L)	0.036 (−0.014–0.085)	0.154 (ns)
M (×10^9^/L)	−0.005 (−0.054–0.045)	0.855 (ns)
PLT (×10^9^/L)	−0.016 (−0.065–0.034)	0.532 (ns)

BMI, body mass index; WC, waist circumference; ASCVD, atherosclerotic cardiovascular disease risk score; SBP, systolic blood pressure; DBP, diastolic blood pressure; ALT, alanine aminotransferase; AST, aspartate aminotransferase; GGT, gamma-glutamyl transferase; hs-CRP, high-sensitivity C-reactive protein; TotCh, total cholesterol; HDL-C, high density lipoprotein cholesterol; TG, triglyceride; LDL-C, low density lipoprotein cholesterol; HbA1c, glycosylated hemoglobin; HOMA-IR, homeostatic model assessment for insulin resistance; WBC, white blood cells; N, neutrophils; L, lymphocytes; M, monocytes; PLT, platelets; TSH, thyroid-stimulating hormone. ns = not significant.

**Table 3:** Multiple regression analysis for TSH levels in subjects at intermediate/high ASCVD risk score.

Predictors	St Coeff	t-Value	*p*
Female sex	0.14	3.6	<0.001
Log-HOMA	0.7	1.9	≤0.05
Log-AST	0.11	2.8	<0.01
Smoking habit	−0.03	−0.8	ns

ns = not significant.

**Table 4:** Multiple regression analysis for TSH levels in subjects with WC > 102 cm M, >88 cm F.

Predictors	St Coeff	t-Value	*p*
Female sex	0.06	2.2	<0.05
Log-HOMA	0.08	2.7	<0.01
Log-AST	0.05	1.7	=0.08
Smoking habit	−0.07	−2.3	<0.05

## Data Availability

The raw data supporting the conclusions of this article will be made available by the authors on request.
